# Siwei Jianbu decoction improves painful paclitaxel-induced peripheral neuropathy in mouse model by modulating the NF-κB and MAPK signaling pathways

**DOI:** 10.1051/rmr/200001

**Published:** 2020-10-20

**Authors:** Jinshuai Suo, Man Wang, Peng Zhang, Yuting Lu, Rong Xu, Ling Zhang, Siyan Qiu, Qiuyan Zhang, Yangyan Qian, Jing Meng, Jing Zhu

**Affiliations:** 1 Jiangsu Key Laboratory for Pharmacology and Safety Evaluation of Chinese Materia Medica, Department of pharmacy, Nanjing University of Chinese Medicine Nanjing China; 2 Department of Neurology and Neuroscience, Johns Hopkins School of Medicine Baltimore USA

**Keywords:** Siwei Jianbu decoction, paclitaxel, peripheral neuropathic pain, MAPK signaling pathway, NF-κB

## Abstract

*Background:* Paclitaxel, a commonly used chemotherapeutic agent, is usually associated with peripheral neuropathy. Paclitaxel induced peripheral neuropathy (PIPN) can be dose limiting and may have detrimental influence on patients' quality of life. However, the mechanism of PIPN remains unclear. Medicinal herbs and their formulas might offer neuronal protection with their multitarget and integrated benefits in chemotherapy-induced peripheral neuropathy (CIPN). Siwei Jianbu decoction (J12) is a classic formula of traditional Chinese medicine which can promote blood circulation and treat diabetic nephropathy in clinical with the symptoms of weakness and pain. *Methods:* The effects of J12 were treated in C57BL/6 mice before injected with Paclitaxel.Behaviour studies: Measurement of mechanical hyperalgesia, thermal nociception and cold allodynia. On the last day at the end of week 6, DRGs were obtained from mice for western blot and immunohistochemical analysis containing NF-κB, p-ERK1/2 and p-SAPK/JNK protein expression. Quantitative real-time polymerase chain reaction: mRNA expression of NF-κB, IL-1β and TNF-α was analyzed. Additionally, the blood samples collected from the eye socket of the mouse were prepared to examine the levels of NF-κB, TNF-α, IL-6 and IL-1β using ELISA assay kits. *Results:* Hypersensitivity tests and pathology analysis have demonstrated that J12 could improve paclitaxel-induced peripheral pain. J12 acts by inhibiting the activation of (C-Jun N-terminal kinases) JNK, (extracellular signal-regulated kinase) ERK1/2 phosphorylation in (Mitogen-activated protein kinases) MAPK signaling pathway and the nuclear factor-κB (NF-κB) in C57BL/6 mice model, J12 also inhibits the production of inflammatory cytokines including tumor necrosis factor α (TNF-α), interleukin 1β (IL-1β) and IL-6. *Conclusion:* The present study showed that J12 ameliorates paclitaxel-induced peripheral neuropathic pain.

## 1 Introduction

Chemotherapy-induced peripheral neuropathy (CIPN) is the major dose-limiting toxicity associated with chemotherapeutic agents such as paclitaxel, oxaliplatin and vincristine [1]. Paclitaxel stabilizes microtubules, which is commonly used to treat breast cancer, ovarian cancer and cervical cancer [2]. However, patients treated with paclitaxel often suffer from paraesthesia, allodynia and hyperalgesia such as tingling and numbness in hands and feet, severe pain is a serious problem for many patients. In addition, CIPN frequently persists long after completion of chemotherapy, thereby reducing quality of life of cancer survivors [3]. Unfortunately, no FDA-approved drugs can prevent or treat chemotherapy-induced peripheral neuropathy.

Neuroinflammation is shown to be involved in several neuropathic pain models [4,5]. Chemotherapy agents can accumulate in the dorsal root ganglia (DRG) [6] which is thought to be used for the transmission of nociceptive stimulation [7]. Recent studies have demonstrated pro-inflammatory immune responses play an important role in chemotherapy-induced peripheral neuropathic pain [8,9]. MAPK pathways including (c-Jun NH2-terminal kinases) JNK, (extracellular signal-regulated kinase) ERK1/2 and p38 contribute to CIPN [10,11]. Furthermore, studies show that paclitaxel activates NF-κB and MAPKs [12]. These pathways induce the expression of phospho-JNK (p-JNK) and phospho-ERK1/2 (p-ERK1/2) in the dorsal root ganglion and the spinal cord with the occurrence of noxious stimuli [13]. Moreover, evidence suggests that paclitaxel treatment can lead to pain sensitivity with the release of proinflammatory cytokine including TNF-α, IL-1β and IL-6 which directly or indirectly induce the neuropathic pain [14].

Siwei Jianbu decoction (J12) is a common clinical medicine which is proved to improve neurological function and symptoms of pain and edema of lower limbs [15]. The formula contains four Chinese herbs: radix paeoniae rubra (ranunculaceae), salvia miltiorrhiza bunge (labiatae), achyranthes bidentata blume (amaranthaceae) and dendrobium nobile (orchidaceae). It is often used to treat diabetic peripheral neuritis, lower extremity thrombosis and chronic kidney disease. The formula has been used to treat 32 kinds of diseases and diabetes is the main disease clinically [16]. However, the therapeutic mechanism of J12 is unclear and studies about analgesic effects of J12 on paclitaxel-induced peripheral neuropathic pain are few.

Therefore, we aimed to clarify the effects of J12 on paclitaxel-induced peripheral neuropathic pain and whether MAPK and NF-κB signal pathways induced by paclitaxel are associated with the preventive impact of J12 on PIPN.

## 2 Materials and methods

### 2.1 Animals

Male C57BL/6 mice weighing 18–22 g (6–8 weeks) were used for these experiments (Nanjing, QingLongShan, China). Free food and water were available. The animals were housed in a room with a normal 12 h light-dark cycle. All animals were habituated in the room for a week before experiments. All experiments were performed in accordance with protocols approved by the Animal Care and Use Committee at the Nanjing University of Chinese Medicine (Approval number AUC171001). All experiments were tested in a blinded manner.

### 2.2 Drugs

Paclitaxel (MKBT3791V, Sigma, USA,) was dissolved with Cremophor EL and ethanol in the radio of 1:1 at concentration of 6 mg/mL and then diluted to 2 mg/mL with 0.9% sterile saline. J12, containing four Chinese herbs, was purchased from Jiangsu Provincial Hospital (Nanjing, Jiangsu, China) and deposited in Nanjing University of Chinese Medicine. The component herbs were decocted twice, each for 1 h. The decoction was filtered and the filtrates were combined and concentrated by rotary evaporation under reduced pressure to 120 mL which is equivalent to 1 g/mL of the original drug.

### 2.3 Paclitaxel-induced neuropathy model

Mice were randomly divided into four groups (*n* = 10 per group). Mice were treated with paclitaxel intraperitoneally (i.p.) at a dosage of 20 mg/kg every other day after two weeks (days 15, 17, 19, 21; cumulative dose 80 mg/kg) to model paclitaxel-induced peripheral pain. Control mice were injected with vehicle only. There were two J12 treatment groups with different dosages of 5 g/kg (J12L) and 10 g/kg (J12H). J12 was injected i.g. daily. It was administered 1 hour before an injection of paclitaxel in the treated groups.

### 2.4 Behavior studies

The behavior test was measured after drug injection weekly and conducted at room temperature. The investigator was blinded to the treatment groups (*n* = 10).

#### 2.4.1 Measurement of mechanical hyperalgesia

To measure mechanical hyperalgesia, we used the Dynamic Plantar Aesthesiometer (DPA, Ugo Basile, Italy). Mice were placed in a plastic chamber to acclimate for half an hour prior to the test. Force was transferred to the hind paw at a frequency of 1 g/s. To minimize the damage, the cut-off force of 10 g was set. The nociceptive threshold was measured when paw withdrawal occurred. This was accessed on each hind paw three trials in total per mouse.

#### 2.4.2 Measurement of thermal nociception

The thermal withdrawal thresholds were assessed on a plantar test (37370, Ugo Basile Plantar Test Apparatus, Italy). Mice were allowed to acclimate in the glass floor for 30 minutes before the test. An infrared source was located at the center of the hind paw of mice, which can transfer heat rapidly. The time of withdrawal was recorded when the source of heat was switched off. In order to avoid injury, a cut-off period of 20 s was maintained. The thermal withdrawal threshold was determined by the average of three treatments per mouse.

#### 2.4.3 Measurement of cold allodynia

The mouse was placed in a fixing apparatus with the tail exposed outside only. The tail of the mouse was immersed in a water bath maintained at 4 °C until tail withdrawal. To avoid damage to the tail, the cut-off time of 20 s was set. The behavior test was repeated three times each mouse with a time interval of at least 15 minutes between two measurements.

### 2.5 Western blot analysis

On the last day at the end of week 6 DRGs were removed from mice for western blot analysis. DRGs were homogenized in RIPA buffer and PMSF (a protease inhibitor) with a ratio of 100:1. Then homogenates were centrifuged at 12,000 rpm for 15 min at 4 °C. Using a BCA protein assay kit, the supernates of protein were collected to be measured. Protein (20 µg) were separated by 10% SDS polyacrylamide gels and transferred onto PVDF membranes in transfer buffer for 60 min. The membranes were soaked in Tris-buffered saline and tween-20 (TBST) containing 5% defatted milk for 60 min at room temperature. They were incubated with rabbit polyclonal antibody against NF-κB (#ab16502,1:2,000; Abcam, MA,UK), p-ERK1/2 (#9101S,1:2,000; Cell Signaling Technology, Danvers, MA, USA), ERK1/2 (#ab17942,1:2,000; Abcam, UK), p-SAPK/JNK (#9251S,1:2,000; Cell Signaling Technology, USA), SAPK/JNK (#8690S,1:2,000; Cell Signaling Technology, USA) and mouse monoclonal antibody GAPDH (#60004-1,1:8,000; Proteintech, China) overnight at 4 °C. The membranes were then washed in TBST three times and incubated with horseradish peroxidase-conjugated goat anti-rabbit IgG (#14708,1:10,000; Cell Signaling Technology, USA) or horseradish peroxidase-conjugated goat anti-mouse IgG (#4416,1:10,000; Cell Signaling Technology, USA) for 60 min at room temperature. The membranes were washed in TBST three times again and the bands were detected by enhanced chemiluminescence. The gray values were quantified using image J software.

### 2.6 Immunohistochemistry

On the last day at the end of week 6, the L4 and L5 DRG were obtained from mice for immunohistochemical analysis. They were fixed in 4% paraformaldehyde overnight and then dehydrated with 30% sucrose solution. The DRGs were cut in 10 µm thickness and mounted on the glass slides. After treating with 0.5% TritonX-100 in PBS for 20 min and then washed with PBS three times quickly, the sections were blocked in 5% normal goat serum and 0.5% Tween-20 in PBS for 1 h at room temperature. The sections were washed with PBS three times again and incubated in primary antibodies containing NF-κB (1:1,000; Abcam), p-ERK1/2 (1:1,000; Cell Signaling Technology) and p-SAPK/JNK (1:1,000; Cell Signaling Technology) overnight at 4 °C. After washing with PBS three times, the sections were incubated in FITC-conjugated secondary antibodies (1:100) for 1 h in the dark at room temperature. Then the sections were washed three times and stained with DAPI (#C0060, Solarbio, China). The immunostained DRGs were viewed under inverted fluorescence microscope.

### 2.7 Quantitative real-time polymerase chain reaction (PCR)

Total RNA was extracted from DRG tissues using TRIzol (Invitrogen, Carlsbad, CA, USA). RNA was used for the synthesis of cDNA with ReverTra Ace qPCR RT Master Mix with gDNA Remover (TOYOBO CO. LTD. Life Science Department, Japan). Real-time PCR was conducted with an Applied Biosystems 7500 RealTime PCR System (Life Technologies, USA) using TransStart Top Green qPCR SuperMix (TransGen Biotech, Beijing, China). GAPDH was used as internal reference and primers were synthesized by Shanghai Sangon Biotech. The primer sequences were as follows: GAPDH, (forward) 5'-GGT TGT CTC CTG CGA CTT CA-3', (reverse) 5'-TGG TCC AGG GTT TCT TAC TCC-3'; NF-κB, (forward) 5'-CTG GTG CAT TCT GAC CTT GC-3', (reverse) 5'-GGT CCA TCT CCT TGG TCT GC-3'; IL-1β, (forward) 5'-TTC AGG CAG GCA GTA TCA CTC ATT G-3', (reverse) 5'-ACA CCA GCA GGT TAT CAT CAT CAT CC-3'; TNF-α, (forward) 5'-GCG ACG TGG AAC TGG CAG AAG-3', (reverse) 5'-GAA TGA GAA GAG GCT GAG ACA TAG GC-3'. In all cases, the effectiveness of amplification was determined by the presence of a single peak in the melting temperature analysis and linear amplification throughout the PCR cycles. 2^−△△Ct^ relative quantification method was used to calculate the relative mRNA impression of the target genes.

### 2.8 ELISA measurements

The blood samples collected from the eye socket of the mouse were prepared to examine the levels of NF-κB, TNF-α, IL-6 and IL-1β using ELISA assay kits (NF-κB, MBE10044; TNF-α, MBE10037; IL-6, MBE10288; IL-1β, MBE10289) according to protocols. Briefly, the supernatants of blood samples were collected after centrifuged at 3000 × *g* for 15 min. The diluted samples and standard solutions were distributed in polystyrene 96-well plates. After incubation for 1h at 37 °C, plates were washed for 5 times. Then color development reagent was added to incubate for 15 min at 37 °C in dark. Stop solution was added to each well. After that, absorbance was recorded at 450 nm within 15 min.

### 2.9 Statistical analysis

All of the experimental data were expressed as means ± S.E.M and analyzed with GraphPad Prism 5. The differences between groups were analyzed by two-way ANOVA followed by Bonferroni post-tests. *P* < 0.05 was considered statistically significant.

## 3 Results

### 3.1 J12 did not change paclitaxel-induced body weight

**Fig. 1:**
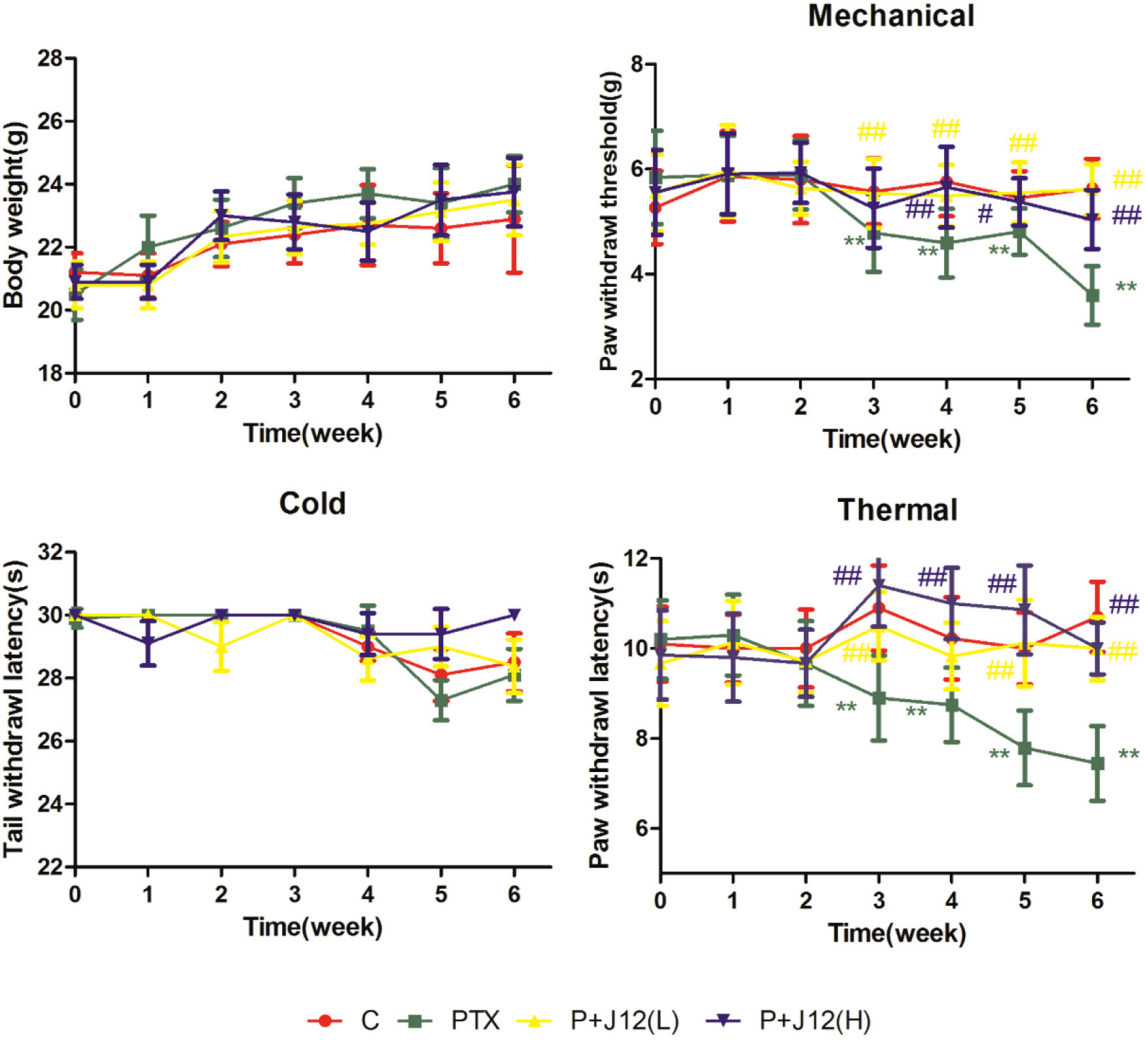
J12 was pretreated 2 weeks before PTX administration. (A) The effect of J12 on body weight in mice treated with or without PTX. No significant difference on model group and J12 treated group in mice. (B) Paw withdrawal threshold was significantly decreased at week 3 and maintained for more than 4 weeks in mice treated with PTX. (C) PTX did not have significant effect on cold tail withdrawal latency. (D) J12 at dosages of 5 g/kg and 10 g/kg both attenuated paclitaxel-induced mechanical hyperalgesia and thermal nociception. Similarly, no effect is observed with J12 on mice treated with or without PTX. **vs Control, *p* < 0.01; # vs PTX, *p* < 0.05; ## vs PTX, *p* < 0.01.

To examine whether J12 had effect on body weight in the paclitaxel-induced neuropathy model, body weight was evaluated in mice after paclitaxel treatment with or without J12. Paclitaxel-induced neuropathy model and drugs administration refer to Supplementary Figure S1. The assessment revealed that group of paclitaxel treatment showed no significant difference in body weight compared to vehicle control mice and administration of J12 did not induce any alteration or enhancement on the physiological body weight compared to model group (Fig. 1 A, *p* > 0.05). Body weights were measured weekly starting from the first injection of J12 through the end of the experiment.

### 3.2 J12 prevented paclitaxel-induced pain hypersensitivity in the mice

As shown in Figure 1, the mice were injected with J12 daily and pain behavior was assessed at the first day every week. At baseline, there was no significant difference between four groups of mice. Pain behavior tests showed that comparison of paclitaxel treatment group with vehicle-treated mice indicated significantly decreases in the paw withdrawal latency from the third week to the 6th week in mechanical hyperalgesia and thermal nociception tests (Fig. 1B and D). In paclitaxel treated group, paw withdrawal threshold was rapidly decreased from 5.89± 0.66 g before paclitaxel to 4.79 ± 0.74 g at week 3 (*p* < 0.01 vs. vehicle control, *n* = 10 in each group) and persisted until week 6 in mechanical hyperalgesia test. Similarly, in model group, paw withdrawal latency time was significantly decreased from 9.67 ± 0.94 s to 8.90 ± 0.94 s at week 3 (*p* < 0.01 vs. vehicle control, *n* = 10) and to 7.44 ± 0.83 s at week 6 in thermal nociception tests. On the other hand, the paw withdrawal threshold was increased on the mice treated with J12 before paclitaxel compared with only paclitaxel-treated group for mechanical hypersensitivity (Fig.1B) and mice pretreated with J12 whether high or low dose both had higher reaction latency times compared with model group for heat sensitivity (Fig. 1D). On the contrary, mice treated with paclitaxel plus J12 or not had no difference on the tail withdrawal latency times compared with vehicle-treated group in cold allodynia test (Fig. 1C, *p* > 0.05) which indicated that paclitaxel could not induce cold hypersensitivity in the mice.

### 3.3 J12 alleviated paclitaxel-induced NF-κB, phospho-ERK1/2 and JNK protein levels in MAPKs in DRGs

**Fig. 2:**
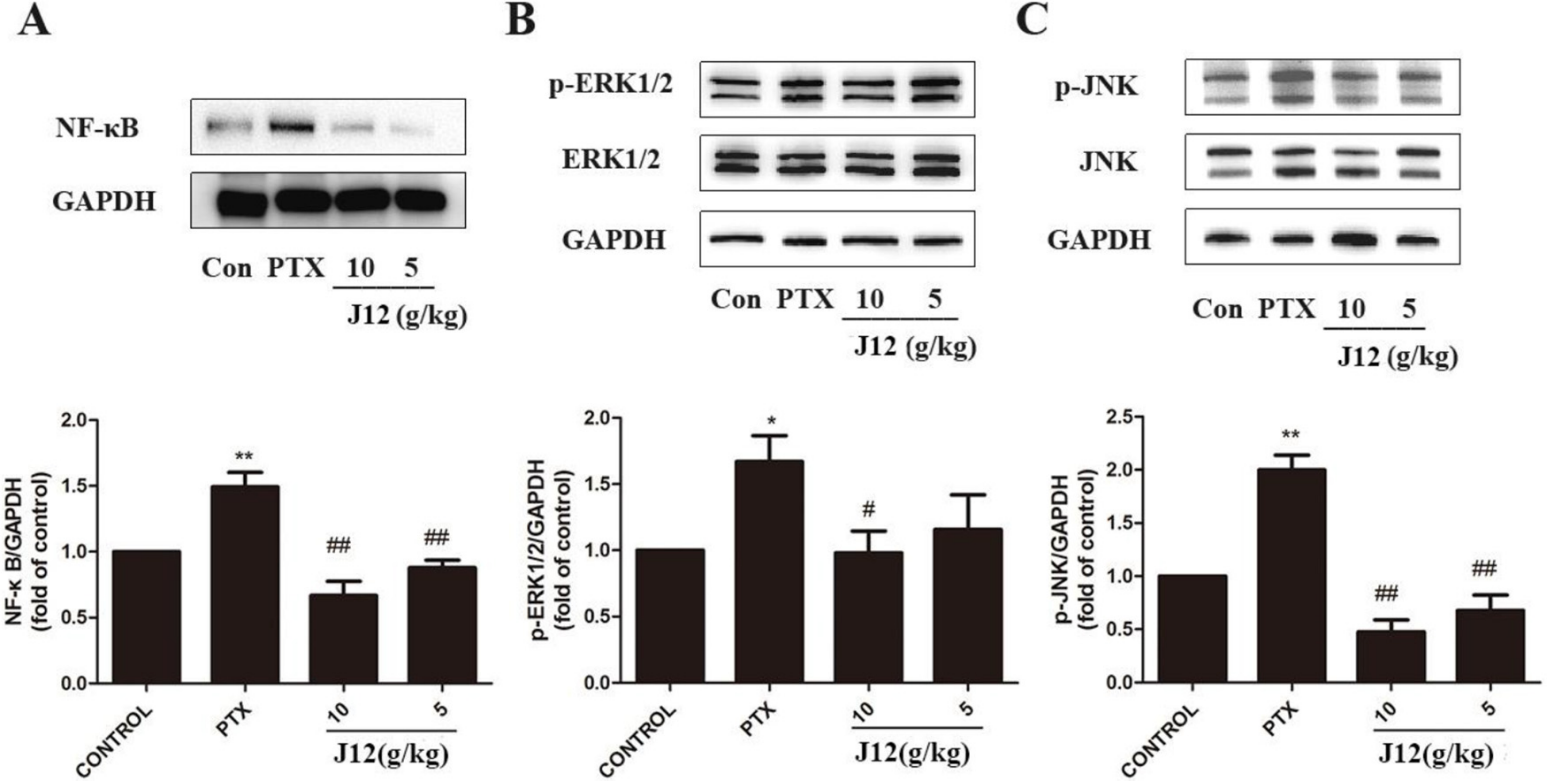
(A) NF-κB was increased in the mice from PTX group and J12 inhibited the activations. (B) p-ERK1/2 was increased in the mice from PTX group and J12 inhibited the activations. (C) p-JNK was increased in the mice from PTX group and J12 inhibited the activations. *n* = 5 in each group. * vs Control, *p* < 0.05; **vs Control, *p* < 0.01; # vs PTX, *p* < 0.05. Data are expressed as mean ± SEM.

**Fig. 3:**
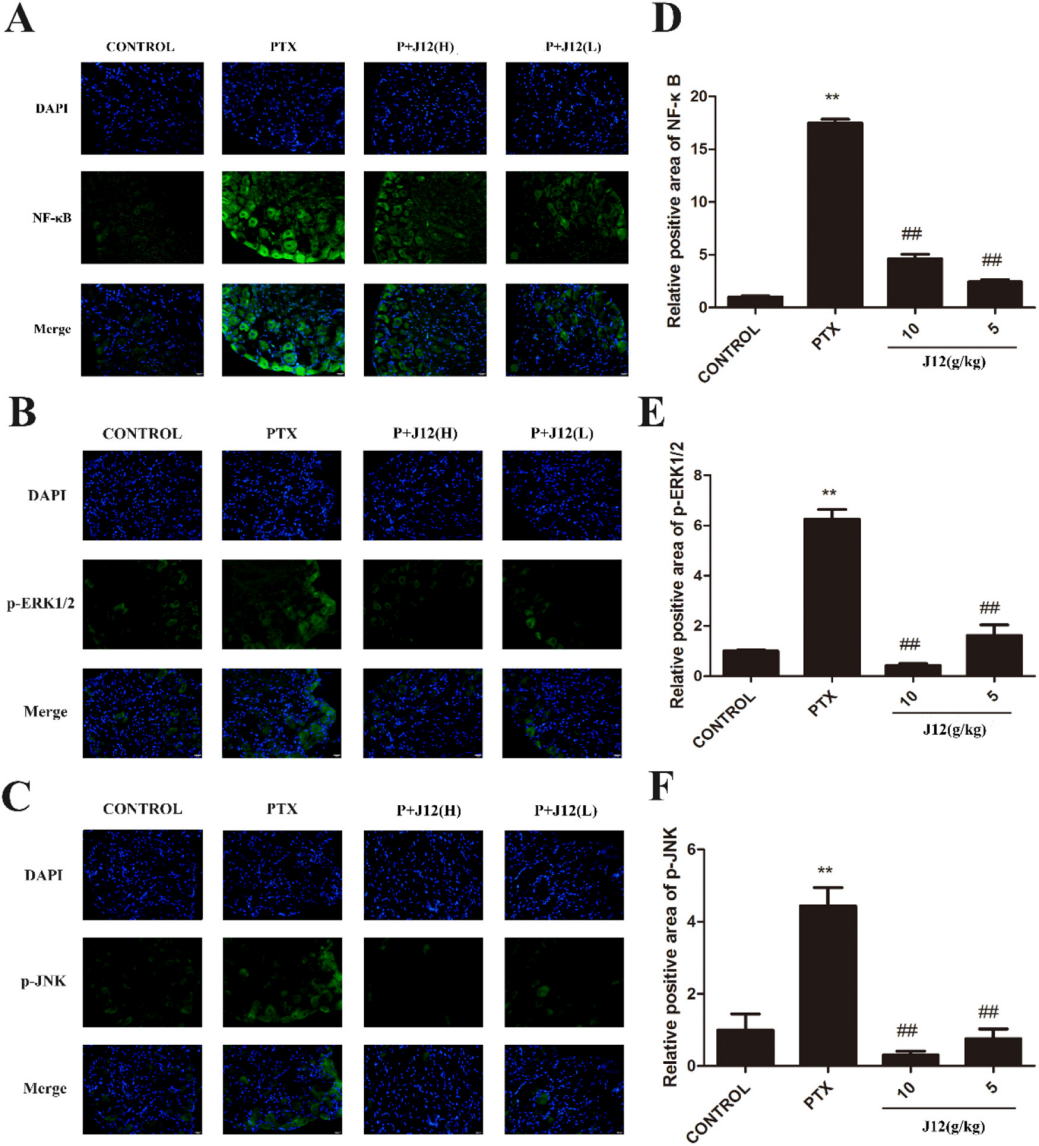
Immunostaining analysis of NF-κB, p-ERK1/2 and p-JNK in DRG neurons. (A–C) The expressions of NF-κB, p-ERK1/2 and p-JNK were increased after PTX treatment and J12 inhibited the expressions. (D) Quantification of NF-κB immunofluorescence intensity in the DRGs. (E) Quantification of p-ERK1/2 immunofluorescence intensity in the DRGs. (F) Quantification of p-JNK immunofluorescence intensity in the DRGs. *n* = 5 in each group. Magnification×400. **vs Control, *p* < 0.01; ## vs PTX, *p* < 0.01. Data are expressed as mean ± SEM.

To investigate the effects of NF-κB and MAPKs signal pathways in PIPN, we examined protein levels in the DRG tissues. Western blotting showed that the expression of NF-κB was increased after paclitaxel treatment (Fig. 2A, *p* < 0.01). Paclitaxel also significantly increased the protein levels of p-ERK1/2 (Fig. 2B, *p* < 0.05) and p-JNK in the DRG tissues compared with vehicle control group (Fig.2C, *p* < 0.01). In addition, at a dose of 10 g/kg or 5 g/kg, J12 inhibited paclitaxel-induced NF-κB, p-ERK1/2 and p-JNK expressions. The injection of a low dose of J12 was more effective for the treatments. We next investigated NF-κB, p-ERK1/2 and p-JNK expressions in the DRG issues using immunostaining (Fig. 3A–C). With image J software analysis, double immunofluorescence data showed that J12 attenuated paclitaxel-induced immunoreactivity compared with the vehicle control group and the levels of protein expressions were the same as western blotting (Fig. 3D–F). The immunoreactive DRG issues are co-localized with DAPI.

### 3.4 J12 inhibited NF-κB and inflammatory cytokines in mice

**Fig. 4:**
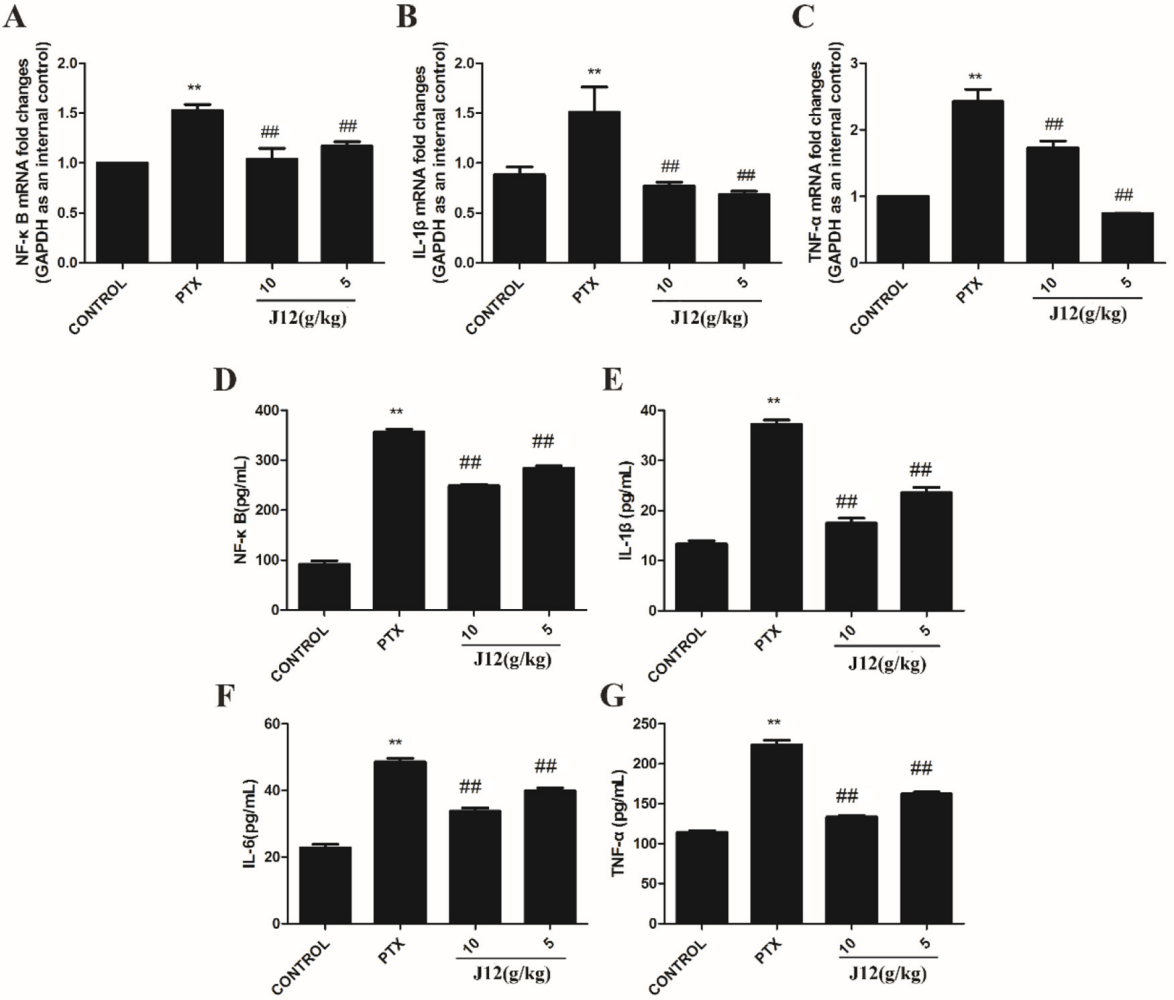
Effect of J12 on paclitaxel-induced NF-κB and inflammatory cytokines in mice. Levels of NF-κB, IL-1β and TNF-α mRNA transcripts were determined using real-time-PCR. (A–C) Treatment with PTX increased the mRNA expressions of NF-κB, IL-1β and TNF-α in DRG neurons as compared with control mice. Injection of J12 attenuated the expressions. Using ELISA kits, we examined levels of NF-κB, TNF-α, IL-6 and IL-1β. (D–G) ELISA analysis showing that J12 attenuated paclitaxel-induced increased expressions. *n* = 5 in each group. **vs Control, *p* < 0.01; ## vs PTX, *p* < 0.01. Data are expressed as mean ± SEM.

With Real-Time PCR assay, we examined the effect of paclitaxel on the levels of NF-κB and pro-inflammatory cytokines including IL-1β and TNF-α in the DRG issues. The data revealed that NF-κB, IL-1β and TNF-α mRNA expressions were increased in paclitaxel-treated mice compared with control group. In addition, J12 attenuated paclitaxel-induced increase in NF-κB, IL-1β and TNF-α mRNA expressions in the DRG issues (Fig. 4A–C). These data suggest that NF-κB and pro-inflammatory cytokine signal pathways are involved in paclitaxel-induced neuropathic pain and J12 can attenuate their upregulation in DRGs. We next examined the expressions with ELISA kits (Fig. 4D–G). ELISA results demonstrated that the expressions of NF-κB, IL-1β, IL-6 and TNF-α were upregulated in paclitaxel-treated mice compared with the mice in control group and J12 attenuated the increase of NF-κB expression and was able to restrain paclitaxel-induced inflammatory cytokines release in mice.

## 4 Discussion

Chemotherapy-induced peripheral neuropathic pain is a major dose-limiting toxicity caused by various types of chemotherapeutic agents. Paclitaxel is widely used as a chemotherapeutic drug for multiple solid tumor. However, paclitaxel-induced peripheral neuropathic pain causes loss of body function, significantly declines the quality of life and even leads to the cessation of treatment. Between 30% and 40% of cancer patients suffer from neuropathic pain [17,18]. Several rodent models have been used to explore the effect of paclitaxel treatment. The administration of paclitaxel frequently results in mechanical allodynia and thermal hyperalgesia [19,20]. Previous evidence revealed that the mice exhibited neuropathic pain symptoms immediately after the injection of paclitaxel and symptoms were able to persist for a long time [21].

Current clinical experience showed that J12 mitigated the neuropathic pain induced by diabetic. Radix paeoniae rubra has an effect on activating blood flow and anticoagulation [22]. Salvia miltiorrhiza bunge from herbs was reported as an antioxidant, anticancer, and anti-inflammatory agent to revert chemotherapy-induced neuropathic pain [23]. Achyranthes bidentata blume is used to protect from cognitive dysfunction and neuroinflammation through modulating ERK pathway and activating NF-κB in animal models [24]. And combination treatment of dendrobium nobile with chemotherapy drug has been shown enhanced anticancer activity through stimulation of JNK stress signaling pathway [25]. But its effect fell short of the complex prescription, the herbs used in coordination can not only obviously strengthen their effect on a variety of symptoms such as reducing nerve injury-related mechanical hypersensitivity and spontaneous pain, restoring lower limb function and other diseases, but also improve the side effect of anti-tumor drugs.

To examine whether J12 is effective for attenuating paclitaxel-induced neuropathic pain including mechanical hyperalgesia and thermal nociception, mice were pretreated with J12 2 weeks that were injected with paclitaxel every other day at week 3. It was demonstrated in our study that J12 significantly increased paw pain thresholds of mice with a dose of 10 g/kg or 5 g/kg and the pain thresholds were similar to those at the baseline. Our results indicate that chronic administration of J12 is effective for ameliorating paclitaxel-induced neuropathic pain.

It has been known that animals treated with paclitaxel caused mechanical hypersensitivity by activating phosphorylated ERK1/2 and JNK in the spinal cord [11]. In addition, it was recently suggested that ERK1/2 was involved in mechanisms of neuropathic pain and the inhibition of JNK reduced apoptosis following paclitaxel treatment [26]. MAPK signaling pathways were shown to play an important role in dorsal root ganglion neurons in CIPN. Moreover, NF-κB is a transcriptional factor which activates in the DRG and spinal cord, and is associated with neuropathic pain [27]. The present study revealed that activation of the NF-κB pathway evoked paclitaxel-induced hyperalgesia [21]. Our study showed that paclitaxel increased the expression of NF-κB in DRG neurons. We also detected increased expression of p-ERK1/2 and p-JNK in DRG with paclitaxel treatment after 6 weeks. However, pre-treatment with J12 was shown to inhibit the levels of NF-κB and phosphorylation of ERK1/2 and JNK. Therefore, we found that J12 exerts an effect of preventing hyperalgesia in paclitaxel-induced neuropathic pain by inhibiting the activation of NF-κB and MAPKs.

It has been reported in previous studies that Inflammatory and peripheral immune responses are involved in chemotherapy-induced peripheral neuropathic pain [28]. Several reports have demonstrated that blockade of the MAPK pathways can be responsible for the central sensitization of inflammatory pain [29]. Studies have shown that NF-κB is observed to regulate the expression of pro-inflammatory cytokines such as TNF-α, IL-1β and IL-6 in DRG and spinal cord in neuropathic pain [30]. In addition, growing evidence supports that paclitaxel treatment induces mRNA expression of the pro-inflammatory cytokines IL-1β and TNF-α and immune cell markers in DRGs [14]. Our current study also showed that after administration of paclitaxel significantly increased the mRNA expressions and serum levels of NF-κB and its downstream cytokines including TNF-α and IL-1β that were prevented by J12 pre-treatment at doses of 5 g kg^−1^ and 10 g kg^−1^ which both had the ability to inhibit increases.

Thus, NF-κB and MAPK signaling pathways play a key role in paclitaxel-induced peripheral neuropathic pain, activated NF-κB contributes to stimulate the release of pro-inflammatory cytokines which generate behavioral hypersensitivity. J12 was demonstrated to have neuroprotective benefits that protected against paclitaxel-induced peripheral neuropathy pain in mice.

## 5 Conclusions

In summary, the current study demonstrated that injection of paclitaxel induced pain hypersensitivity, and J12 exerts anti-hyperalgesic effect on mechanical and heat hyperalgesia in mice by suppressing paclitaxel-induced activation of NF-κB and pro-inflammatory cytokines. Also, J12 alleviates neuropathic pain by blocking ERK and JNK pathways. Therefore, J12 has potential as new therapeutic agent targeting NF-κB and MAPK signaling pathway to ameliorate paclitaxel-induced peripheral neuropathic pain and decrease adverse reaction of anti-tumor drugs.

## Conflicts of Interest

The authors declare that they have no conflicts of interest.

## Supplementary Material

Figure S1. Paclitaxel-induced neuropathy model and drugs administration. The Supplementary Material is available at https://doi.org/10.1051/rmr/200001.
