# Supplementary figures and images for: Siwei Jianbu decoction improves painful paclitaxel-induced peripheral neuropathy in mouse model by modulating the NF-κB and MAPK signaling pathways
Source: Regen Med Res. 2020 Oct 20;8:2. doi: 10.1051/rmr/200001 (PMC7583579; doi:10.1051/rmr/200001)

Supplementary data

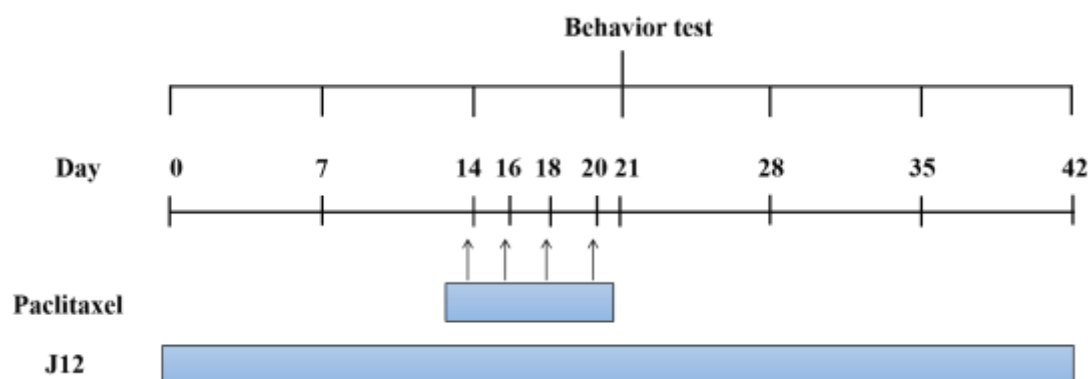

Figure S1. Paclitaxel-induced neuropathy model and drugs administration.

Supplement: Supplementary file 1 — Figure S1. Paclitaxel-induced neuropathy model and drugs administration. [file rmr-8-2-s1.pdf]
